# Thermoregulatory effects of swaddling in Mongolia: a randomised controlled study

**DOI:** 10.1136/archdischild-2014-307908

**Published:** 2015-10-29

**Authors:** Bazarragchaa Tsogt, Semira Manaseki-Holland, Jon Pollock, Peter S Blair, Peter Fleming

**Affiliations:** 1 Public Health Institute of Mongolia, Ulaanbaatar, Mongolia; 2 Public Health, Epidemiology & Biostatistics, University of Birmingham, Birmingham, UK; 3 Faculty of Health & Applied Sciences, University of the West of England, Bristol, UK; 4 School of Social and Community Medicine, University of Bristol, Bristol, UK

**Keywords:** Epidemiology, Comm Child Health, Temp Regulation

## Abstract

**Objective:**

To investigate thermal balance of infants in a Mongolian winter, and compare the effects of traditional swaddling with an infant sleeping-bag in apartments or traditional tents (Gers).

**Design:**

A substudy within a randomised controlled trial.

**Setting:**

Community in Ulaanbaatar, Mongolia.

**Subjects:**

A stratified randomly selected sample of 40 swaddled and 40 non-swaddled infants recruited within 48 h of birth.

**Intervention:**

Sleeping-bags and baby outfits of total thermal resistance equivalent to that of swaddled babies.

**Outcome measure:**

Digital recordings of infants’ core, peripheral, environmental and microenvironmental temperatures at 30-s intervals over 24 h at ages 1 month and 3 months.

**Results:**

In Gers, indoor temperatures varied greatly (<0–>25°C), but remained between 20°C and 22°C, in apartments. Despite this, heavy wrapping, bed sharing and partial head covering, infant core and peripheral temperatures were similar and no infants showed evidence of significant heat or cold stress whether they were swaddled or in sleeping-bags. At 3 months, infants in sleeping-bags showed the ‘mature’ diurnal pattern of a fall in core temperature after sleep onset, accompanied by a rise in peripheral temperature, with a reverse pattern later in the night, just before awakening. This pattern was not related to room temperature, and was absent in the swaddled infants, suggesting that the mature diurnal pattern may develop later in them.

**Conclusions:**

No evidence of cold stress was found. Swaddling had no identifiable thermal advantages over sleeping-bags during the coldest times, and in centrally heated apartments could contribute to the risk of overheating during the daytime.

**Trial registration number:**

ISRTN01992617.

**What is already known on this topic**

- Swaddling is widely used in many societies, but little is known about optimal levels of wrapping or the effects on infant thermoregulation.
- Infants can tolerate very wide ranges of added insulation at night, but covering the head can potentially compromise their ability to thermoregulate.
- A number of factors (eg, low birth weight and bottle-feeding) are associated with later development of mature diurnal temperature patterns in infants.

**What this study adds**

- Even in extreme environmental conditions swaddling offers no identifiable thermal advantage over the use of baby sleeping-bags.
- Babies living in Gers with extremes of diurnal temperature variation who used sleeping-bags developed mature diurnal temperature patterns earlier than those who were swaddled.
- Swaddled infants showed effective thermoregulation despite very high levels of added insulation, including head covering.

## Background

Swaddling the baby in the cold season is a traditional practice in Mongolia and normally involves tight, prolonged wrapping from the head or neck down in two to three layers of thin cotton cloth, covered by layers of thick blankets or sheepskin (see online supplementary appendix IA). For the first 3 months, the baby is swaddled most of the day and night, with a gradual decrease in intensity and duration of wrapping until beyond 6 months.

Mongolia is a landlocked, East-Asian, developing country of high literacy (>90%) and with a population of 2.7 million people, 32% of whom live below the poverty line.^1^ ^2^ The climate is dry with extreme temperatures ranging from −40°C in the long winter to +30°C in the summers in Ulaanbaatar.

Swaddling is being promoted in many Western countries^3^ ^4^ as a method of sleep promotion and ensuring supine sleep, while it is practised in many temperate Far-Eastern, Middle-Eastern, Central Asian, former Soviet and South American countries^4^ for practical childcare reasons, protection from cold, and a belief in a number of effects including the promotion of sleep and calm in babies. In Mongolian winter, given the low temperatures, swaddling could be important to protect infants from cold stress which is a risk factor for respiratory infection,^5^ but little is known of any effects of swaddling on thermoregulation in infancy in any environmental condition. This could be important in many ways including as a contributor to Sudden Infant Death Syndrome (SIDS) or respiratory infections in infants.

The majority of the population in Mongolia and almost half of the capital city Ulaanbaatar's one million residents occupy traditional dwellings, the ‘Ger’ (see online supplementary appendix IC). Temperatures within the Ger vary widely in the winter, being very warm in the daytime and very cold at night. Other residents of Ulaanbaatar live in relatively modern centrally heated apartments, connected to a centralised heating system that maintains relatively constant indoor temperatures.

We investigated in a randomised controlled trial (RCT) the effect of swaddling on a range of thermal recordings during a Mongolian winter, and compared the effects of swaddling with a combination of clothing and sleeping-bags designed to be of equal thermal resistance. A secondary objective was to investigate the effect of housing type (and therefore environmental temperature variations) upon this effect.

## Methods

### Sample

**Figure 1:**
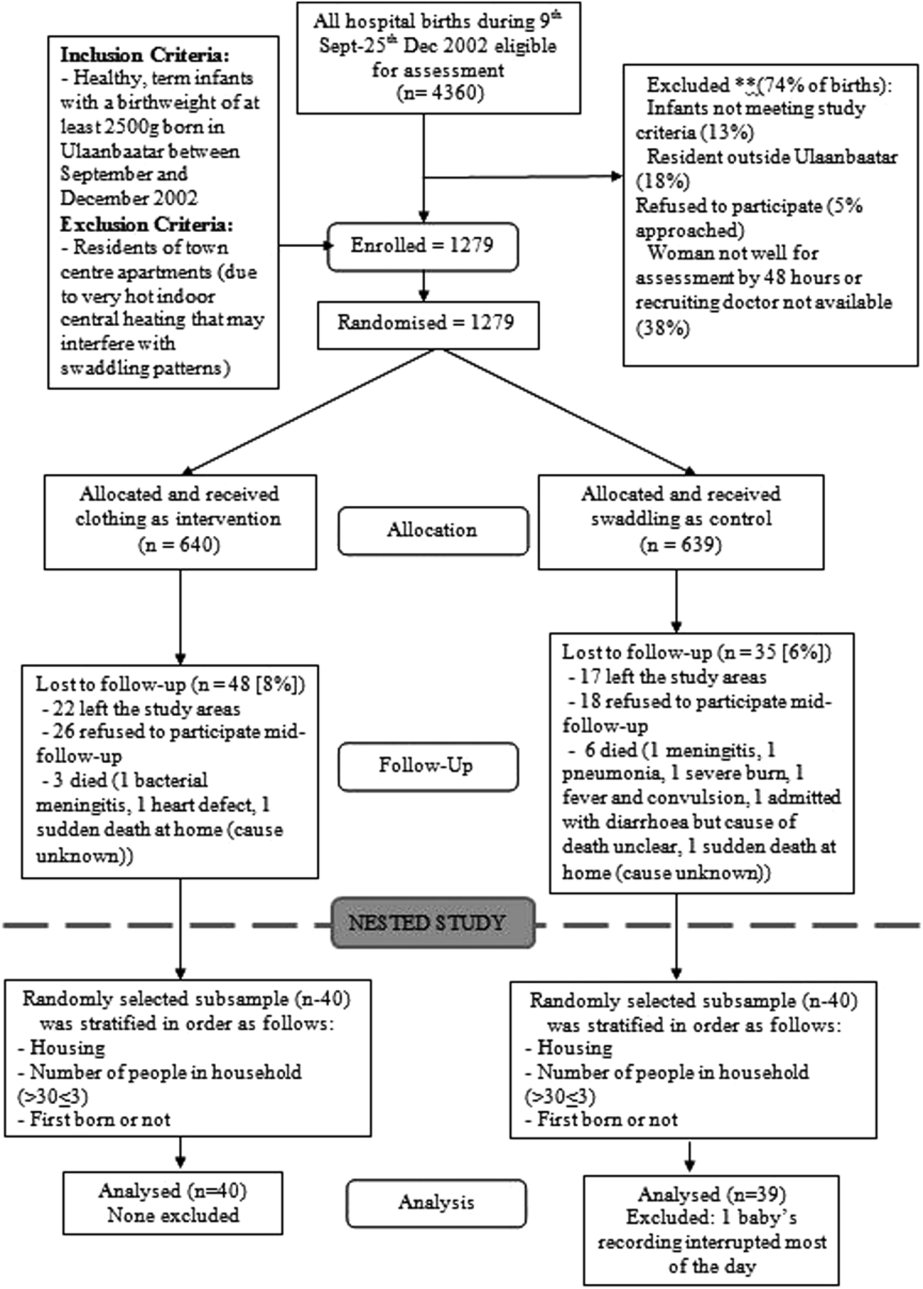
The trial flow chart.

This study was nested in a larger prospective RCT designed to investigate the effects of traditional swaddling on pneumonia, health, growth and development of infants in Mongolia.^6^ The main study involved 1274 healthy infants ≥37 weeks gestation and a birth weight of >2500 g, born in Ulaanbaatar between September and December 2002. All healthy infants delivered at the only four maternity hospitals in Ulaanbaatar, Mongolia were eligible to be recruited within 48 h of birth (figure 1). More than 95% of Ulaanbaatar births took place in these four hospitals.^7^ The residents of the town centre apartments known to have very hot indoor central heating were excluded as qualitative work indicated that this interfered with their swaddling patterns. Infants were randomly allocated to swaddled or non-swaddled groups within 48 h of birth. The two arms had comparable characteristics and randomisation was successful (figure 1).

For the present nested study of detailed recordings, in the peak of winter during December 2002 and March 2003 (therefore selecting babies in November and December), we randomly selected a subset of 40 healthy infants from each arm of the main study stratified by type of dwelling, the number of people living in the household and birth order. After 16 strata were defined, an Excel random number generated list was applied to each stratum and three from each stratum were selected. The infants were those fully compliant to the intervention regimen.

### Sample size calculation

There is little information on the body temperature of swaddled infants in cold environments from previous studies on which to base sample size calculations. The anticipated size of the difference to detect in abdominal skin (core) temperatures between swaddled and infants in sleeping-bags was estimated at 0.5°C,^8–10^ and the lower and upper ranges of normal abdominal skin temperatures as 36°C and 37°C, respectively, were used.^11^ To detect a 0.5°C difference with 95% confidence at the 5% significance level with 80% power the fundamental equation was used. This suggested using at least 17 infants per group or 34 infants as the minimum study sample size. Assuming a 20% dropout or refusal, 42 infants would be required. However, during the actual study period from 3 December to 26 December, 238 infants reached 1 month of age. Within the resources available, the investigator nearly doubled the sample size of the study to improve estimates and representation of the targeted population.

### Intervention

Families in both groups were asked to cover the baby as they felt was appropriate for the environmental temperature.^6^ Among the swaddled babies (control arm of the trial) the families mostly followed the traditional practice, using cotton and woollen wrapping materials/blankets, together with sheepskins on occasions while in the intervention group they used their own clothes, additional baby clothes and baby sleeping-bags provided for them by the study since non-swaddling options in shops were scarce (see online supplementary appendix 1B). As explained elsewhere, blinding was not possible in such interventions, but thorough compliance monitoring/data is available for each child.^6^ The analysis was blind to allocation.

### Measurement of thermoregulation

Digital recordings of infants’ core, peripheral, environmental and microenvironmental temperatures were made on two occasions in the infant's home, each over a 24 h period: (1) at 1 month and (2) at 3 months of age during the coldest months in the Mongolian year (December–March).

Mothers recorded detailed logs of infant activity and wrapping (including bed sharing adult blankets over the babies at nights) during the recording period using a pretested 24-h diary. They were asked not to bathe the babies in this 24 h due to the skin probes. Because of the social unacceptability of rectal probes in young infants in Mongolia, we recorded core temperatures using abdominal skin temperature probes over the liver, with insulation over the probe on the surface away from the infant (previously shown to reliably track core temperature).^8^ ^12^ For the purposes of comparisons between infants and between sleeping conditions, temperature data were averaged over 15 min periods, each consisting of 30 recorded observations. For the purpose of comparisons between day and night time data we have designated daytime as 9:00 to 21:00, and night-time as 21:00 to 9:00. A temperature data logger (Squirrel) recording at 30 s intervals on four channels was used to simultaneously record the core body and peripheral (skin of anterior shin)^8^ temperatures, the room temperature (outside all clothing or bedding, but close to infant) and the microenvironmental temperatures in the infant's bedding (inside the sleeping-bag or outer cover or layers of swaddling, but outside the first layers of baby clothing).^12^

### Assessment of thermal resistance (‘tog values’) of bedding and clothing

Samples were collected of a range of the typical swaddling and clothing materials used and of the infant sleeping-bags produced for the study. At the end of the study direct measurements of the thermal resistance (in tog units) of non-compressed samples of each of these materials were made in the Performance Clothing laboratories of the School of Design in the University of Leeds (Dr David Brook).

We have assumed that the laboratory measurement of thermal resistance is the effective insulation value of each material when in use. In practice the effect of trapped air between layers of bedding or clothing is to increase the insulation values of combinations of materials, but as this variable could not be measured and is likely to be similar between groups we have not taken it into account in our calculations of total insulation levels.

Thermal resistance was calculated using these laboratory-measured values applied to the detailed maternal logs of the infants’ bedding and clothing materials (including head covering). The total effective insulation (in tog units) was calculated by summing the individual contributions from each item of clothing/swaddling and bedding applied, corrected for the proportion of the surface area of the baby that the item covered.^8^ ^12^ ^13^

### Statistical analysis

The number of detailed recordings pragmatically possible to collect in such studies is unavoidably limited. We reduced the impact of skewed data by adopting a non-parametrical approach to the analysis. The Mann-Whitney U test (two-sided) was used to compare the differences between distributions.

The χ^2^ test for independence with Yates’ correction was used to test differences between proportions. If an expected cell was below 5 the Fisher's exact test was used. Spearman's rank order correlation (r) was used to calculate the strength of the relationship between continuous variables.^14^

## Results

### Background infants’ characteristics

Table 1 describes the family and child characteristics of the sample between 39 swaddled and 40 non-swaddled infants which were equally distributed between the arms. Background characteristics of the sample were comparable with the main study (data not shown). The stratification was successful with no significant differences identified between infants in the swaddling group and the sleeping-bag group for the type of dwelling, number of people in the household or whether the infant was first-born or not. The only baseline variable to significantly differ by chance was birth weight; infants swaddled were slightly but significantly heavier (Mann-Whitney U test: p=0.002).

**Table 1:** Background family and child characteristics; all differences in characteristics between groups are not statistically significant with p values ranging from 0.99 to 0.20, except birth weight where p=0.002

Background characteristics	Swaddling		Sleeping-bag	
	n	Per cent	n	Per cent
*Total included in analysis*	*39*	*100*.*0*	*40*	*100*.*0*
Type of dwelling				
Ger	20	(52.3)	16	(40.0)
Apartment	19	(47.7)	24	(60.0)
Number of people in the household				
≤3 people per room	20	(51.3)	22	(55.0)
>3 people per room	19	(48.7)	18	(45.0)
Birth order				
First	18	(46.2)	19	(48.8)
Subsequent	21	(53.8)	21	(51.2)
Gestational age at birth				
<37 weeks		*–*	*3*	*(4*.*0)*
≥38 weeks	*39*	*(100)*	*37*	*(96*.*0)*
Type of delivery				
Normal vaginal birth	*34*	*(87*.*2)*	*30*	*(75*.*0)*
Caesarean	*3*	*(7*.*7)*	*8*	*(20*.*0)*
Other	*2*	*(5*.*1)*	*2*	*(5*.*0)*
Gender				
Male	17	(42.5)	16	(38.5)
Female	22	(57.5)	24	(61.5)
Breast feeding after birth				
Within 30 min	29	(78.4)	30	(79.0)
Within 30–60 min	6	(13.5)	4	(5.3)
Within 24 h	4	(8.1)	6	(15.7)
Breast feeding at 1 month				
Yes	38	(97.5)	38	(97.8)
No	1	(2.5)	2	(2.2)
Breast feeding at 3 months				
Yes	36	(90.0)	37	(94.9)
No	3	(10.0)	3	(5.1)
Bed sharing at 1 month				
With one adult	30	(79.5)	26	(65)
With two adults	10	(20.5)	14	(35)
Bed sharing at 3 months				
With one adult	26	(66.7)	22	(55)
With two adults	13	(33.3)	18	(45)
Maternal age at recruitment				
20–29 years	26	66.7		
≥30 years	13	33.3		
Maternal marital status				
Married	20	52.6		
Cohabiting	18	47.4		
Maternal education				
Primary and secondary	16	41.0		
Tertiary	23	59.0		
Paternal education				
Primary and secondary	23	62.9		
Tertiary	13	37.1		
Father ever worked				
Yes	31	79.5		
No	8	20.5		
Smokers resident in home				
None	19	48.7		
One or more	21	51.3		

	**Median**	**IQR**	**Median**	**IQR**

Birth weight (kg)	3.6	3.2–3.9	3.3	3.1–3.5
Length (cm)	51	50–52	51	50–52

Bed sharing with parents was assessed on two consecutive nights at 1 month and 3 months of age and found to be a universal practice, with no differences between the groups.

### Room temperature variation

The median room temperatures were recorded for each 30 min period over the 24 h duration of the recordings at the two ages for infants living in Gers and those living in apartments (see online supplementary appendix II).

The room temperature in the apartments varied very little over the 24 h period, staying between 20°C and 22°C for almost the whole time, a similar range to that previously published for infant homes in the UK.^13^ ^15^ In Gers the median room temperatures recorded varied considerably over each 24 h period (from 13°C to 21°C) while individual indoor temperatures ranged from below 0°C in the early morning, rising to above 25°C in the late afternoon (see online supplementary appendix II).

### Thermal insulation

The median total effective insulation levels (tog values) for bedding and clothing during night-time and daytime were consistently higher for swaddled infants than for those in sleeping-bags regardless of whether the infants slept in Gers or apartments (see online supplementary appendix III).

Similar differences were noted at 3 months of age. The total effective insulation from bedding and clothing was higher for swaddled infants during the night than infants in sleeping-bags in both types of dwelling (higher by 3 togs in Gers and 1.9 togs in apartments, Mann-Whitney U test: p<0.0001). The differences in the day time were much smaller and non-significant (0.3 tog difference in Gers).

This total thermal resistance of bedding plus clothing for the swaddled infants being significantly higher than for non-swaddled infants (table 2) at 1 month and 3 months was reflected in the higher microenvironmental temperatures (inside the bedding but outside the clothing) of the swaddled versus non-swaddled infants in both periods (table 2).

**Table 2:** The overall 24-h median temperatures for 1-month-old and 3-month-old infants separately

Temperatures averaged over 24 h (whether living in apartments or Gers)										
One-month-old						Three-months-old				
Temperature/total togs	Swaddled (n=39)		Sleeping-bag (n=40)		Mann-Whitney U test	Swaddled (n=39)		Sleeping-bag (n=40)		Mann-Whitney U test
	Median	IQR	Median	IQR		Median	IQR	Median	IQR	
Room	19.0	(16.8–21.7)	21.1	(18.1–22.4)	0.07	20.5	(18.4–21.6)	20.8	(17.8–22.8)	0.34
Peripheral	34.9	(34.4–35.7)	34.7	(33.5–35.2)	0.17	28.6	(27.2–29.7)	29.3	(27.7–30.4)	0.04
Core	36.9	(36.5–37.1)	36.7	(36.5–36.9)	0.043	36.1	(35.9–36.7)	36.0	(35.7–36.4)	0.06
Microenvironment	35.7	(35.7–35.9)	35.3	(34.7–35.6)	0.008	34.8	(33.9–35.1)	34.3	(33.8–34.8)	0.009

Shading shows statistically significant results.

### Infants’ temperatures at 1 month

No harmful effects resulting from the intervention were detected by the researchers or the trial monitoring committee. Table 3 shows the median values over the 12 h daytime and night-time periods for environmental and infant temperatures of 1-month-old infants in Gers and in apartments, respectively.

**Table 3:** Daytime and night-time median temperatures and total tog measurements of 1-month-old infants in Gers and apartments

One-month-old infants in Gers										
Daytime						Night-time				
Temperature/total togs	Swaddled (n=20)		Sleeping-bag (n=16)		Mann-Whitney U test p value	Swaddled (n=20)		Sleeping-bag (n=16)		Mann-Whitney U test p value
	Median	IQR	Median	IQR		Median	IQR	Median	IQR	
Room	18.7	(17.0–20.1)	18.2	(16.8–19.2)	0.57	15.0	(13.3–17.7)	16.3	(13.5–18.3)	0.67
Peripheral	35.0	(34.3–35.2)	33.7	(32.6–34.5)	<0.001	35.1	(34.8–35.6)	35.0	(33.5–35.4)	0.19
Core	37.0	(36.7–37.1)	36.3	(36.1–36.6)	0.006	36.8	(36.6–37.0)	36.7	(36.3–36.7)	0.05
Microenvironment	35.2	(34.5–35.8)	34.0	(33.3–34.7)	0.009	35.8	(35.0–36.1)	35.7	(34.8–36.1)	0.52
Togs	8.5	(7.6–11.0)	6.4	(6.0–7.2)	<0.0001	13.2	(11.1–14.6)	9.3	(7.3–12.0)	<0.0001

**One-month-old infants in apartments**										
**Daytime**						**Night-time**				
**Temperature/total togs**	**Swaddled (n=19)**		**Sleeping-bag (n=24)**		**Mann-Whitney U test p value**	**Swaddled (n=19)**		**Sleeping-bag (n=24)**		**Mann-Whitney U test p value**
	**Median**	**IQR**	**Median**	**IQR**		**Median**	**IQR**	**Median**	**IQR**	

Room	21.8	(19.2–22.6)	21.8	(20.3–22.7)	0.65	21.0	(20.0–22.5)	21.7	(19.7–23.4)	0.46
Peripheral	33.8	(32.7–34.9)	34.3	(33.3–35.0)	0.40	34.4	(33.8–35.0)	35.1	(34.1–35.4)	0.89
Core	36.7	(36.4–37.0)	36.6	(36.0–36.9)	0.65	36.8	(36.4–37.0)	36.8	(36.5–37.0)	0.17
Microenvironment	35.1	(34.4–35.6)	34.9	(34.2–35.3)	0.12	35.8	(35.5–36.1)	35.5	(35.1–35.9)	0.04
Togs	8.0	(7.0–10.7)	6.5	(5.6–7.9)	<0.0001	13.0	(8.7–14.4)	9.7	(7.9–12.2)	<0.0001

Shading shows statistically significant results.

When viewed over the whole of these 12 h periods, in the night-time, there were no significant differences in median core or peripheral temperatures between swaddled infants and those in sleeping-bags in either Gers or apartments (tables 3 and 4). Overall in 24 h (table 2), the core body temperature was also slightly but significantly higher in the swaddled group at 1 m. Despite these differences, however, the peripheral temperatures of the non-swaddled infants (a marker of peripheral vasoconstriction in response to cold stress) were not lower than those of the swaddled infants, suggesting that both groups of infants were in thermoneutral conditions.

**Table 4:** Daytime and night-time median temperatures of 3-month-old infants in Gers and apartments

Three-month-old infants in Gers										
Daytime						Night-time				
Temperature/total togs	Swaddled (n=20)		Sleeping-bag (n=16)		Mann-Whitney U test p value	Swaddled (n=20)		Sleeping-bag (n=16)		Mann-Whitney U test p value
	Median	IQR	Median	IQR		Median	IQR	Median	IQR	
Room	19.2	(17.5–21.8)	19.2	(18.4–22.9)	0.48	16.6	(12.6–18.6)	17.8	(12.5–19.4)	0.62
Peripheral	28.1	(28.0–30.0)	29.3	(27.4–32.0)	0.58	30.0	(28.4–31.0)	31.0	(30.0–32.0)	0.18
Core	36.3	(36.0–36.5)	36.0	(35.3–36.3)	0.10	36.4	(36.2–36.9)	36.0	(35.5–36.3)	0.02
Microenvironment	34.5	(33.3–34.8)	33.8	(33.7–34.3)	0.20	35.2	(34.9–35.8)	34.9	(34.0–35.2)	0.05
Togs	10	(9.0–11.2)	9.7	(7.1–11.6)	0.12	13.2	(10.4–16.0)	10.2	(9.5–12.0)	<0.0001

**Three-month-old infants in apartments**										
**Daytime**						**Night-time**				
**Temperature/total togs**	**Swaddled (n=19)**		**Sleeping-bag (n=24)**		**Mann-Whitney U test p value**	**Swaddled (n=19)**		**Sleeping-bag (n=24)**		**Mann-Whitney U test p value**
	**Median**	**IQR**	**Median**	**IQR**		**Median**	**IQR**	**Median**	**IQR**	

Room	21.5	(20.1–22.1)	21.7	(20.7–23.3)	0.19	21.3	(20.1–22.1)	22.1	(19.8–22.6)	0.29
Peripheral	29.0	(27.3–29.2)	29.0	(29.0–30.0)	0.09	29.3	(28.0–31.0)	31.1	(29.2–33.0)	0.03
Core	36.0	(35.8–36.4)	36.1	(35.7–36.3)	0.98	36.0	(35.8–36.3)	36.2	(35.6–36.5)	0.83
Microenvironment	34.2	(33.6–34.9)	34.1	(33.0–34.5)	0.33	35.0	(34.8–35.5)	35.0	(34.4–35.2)	0.37
Togs	9.4	(7.9–10.5)	8.9	(6.4–9.7)	<0.0001	11.5	(8.9–13.2)	9.6	(8.9–12.0)	<0.0001

1. Shading shows statistically significant results.

2. Where the total number in the columns does not match the total number shown in the row heading, there were inadequate recordings die to greater activity of 3-month-old infants, thus affecting the sensors.

The only factor that was not balanced between the swaddling and the sleeping-bag groups was the birth weight of the study babies (table 1). Although all of the infants in this study had birth weights of more than 2500 g, swaddled infants were significantly (median 300 g) heavier than the infants in sleeping-bags. Thus, this factor was further taken into account in the analysis. Bivariate correlations between birth weight and key temperatures for all infants (swaddled and sleeping-bag together) were all non-significant. Core and peripheral temperatures (24 h, day or night measurements) assessed against birth weight using Spearman’s correlation coefficients (coefficients between 0.05 and −0.22, with values between 0.21 and 0.77) indicated that birth weight had no demonstrable relationship with infant body temperatures and, consequently, birth weight variation was unlikely to explain any temperature differences determined.

However, the microenvironmental temperatures (ie, temperatures outside the baby's clothing but inside the bedding) were slightly but significantly higher in the apartments for swaddled infants, partly reflecting the higher insulation used for these babies (13 vs 9.7 median tog in apartment).

**Figure 2:**
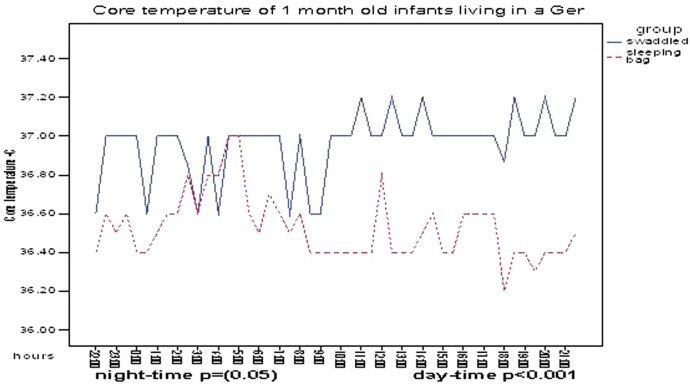
Diurnal core temperature patterns (median values) for swaddled and sleeping-bag infants living in Gers at age 1 month.

When the median temperatures were viewed for each sequential 15 min period, however, a slightly different picture emerged. For much of the night the median core temperatures over this same 24 h period for 1-month-old infants living in Gers (figure 2 and table 3) were higher for swaddled infants than for those in sleeping-bags, and these differences were more marked in the daytime, when peripheral temperatures were also higher. This was not completely explained by the higher thermal insulation (tog values) among swaddled infants as these differences in core temperature were not seen in infants sleeping in apartments despite similar differences in thermal insulation.

### Infants’ temperatures at 3 months

Table 4 shows the median values for environmental and infant temperatures over the whole 12 h daytime and night-time periods at age 3 months in Gers and apartments. As for the results at 1 month, there were no significant differences in room temperature for swaddled infants compared with those in sleeping-bags in Gers or apartments.

In contrast to the findings at 1 month, however, the data from the whole 12 h daytime periods show no significant differences in the median core, peripheral or microenvironmental temperatures between swaddled infants and those in sleeping-bags—either in Gers or in apartments (tables 2 and 4)

At night however, in Gers, the median night-time core temperatures were higher for swaddled infants, while peripheral temperatures were not significantly different. In apartments there was no difference in core temperatures at night, but peripheral temperatures were higher for infants in sleeping-bags.

**Figure 3:**
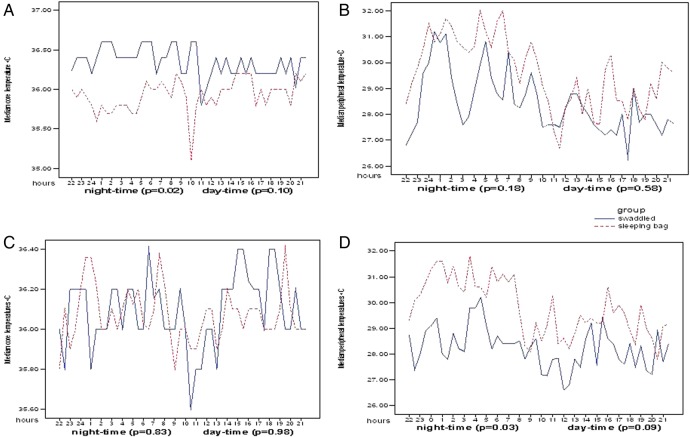
Diurnal core temperatures (median values) for swaddled and sleeping-bag infants in: (A) Gers and (C) apartments at age 3 months; and diurnal peripheral temperatures (median values) for swaddled and sleeping-bag infants in: (B) Gers and (D) apartments at age 3 months.

Figure 3A shows for infants in Gers that when the data are plotted for each consecutive 15 min period during the night, the median core temperature of infants in sleeping-bags fell (from 36.0°C to 35.6°C) around 1 h after babies were put down to sleep and remained below 36°C until shortly before waking at 05:00. An apparent very brief further fall later in the morning—seen in swaddled and sleeping-bag infants—is probably related to the first change of nappy/clothing of infants usually occurring around the same time in most families thus accumulating to a dip in the average of recordings. The overnight fall in core temperature was not correlated with changes in room temperature during this period (Spearman’ r=0.18, p=0.54), but was similar to the pattern previously described in healthy 3–4-month-old infants.^15–19^ Strikingly, this nocturnal fall in core body temperature was absent in swaddled infants.

Figure 3B shows the corresponding diurnal pattern seen when the values of median peripheral temperature from sequential 15 min periods for these infants are plotted. Despite the observed lower core temperature during the night-time hours, for infants in sleeping-bags their peripheral temperatures were consistently slightly higher than for those who were swaddled. This is evidence of peripheral vasodilatation in the infants in sleeping-bags, not vasoconstriction, as would occur if the observed lower core temperatures were a consequence of cold stress.

In contrast, no such differences between core temperatures of swaddled infants and those using sleeping-bags were observed for 3-month-old infants living in apartments (figure 3C). There was no drop in core temperature observed for 3-month-old infants in sleeping-bags living in apartments at nights. However, the peripheral temperatures of these infants were consistently 1–3°C higher than for swaddled infants during the night-time (figure 3D), again showing evidence of peripheral vasodilatation in this group, despite being significantly less heavily wrapped than the swaddled infants.

## Discussion

This study assessed for the first time the effects of traditional swaddling and the use of sleeping-bags on infant thermoregulation in non-laboratory conditions in a country with very cold winter months. Despite harsh environmental conditions, heavy wrapping, bed sharing and partial head covering, no infants showed evidence of significant heat or cold stress whether they were swaddled or in sleeping-bags.

Review of the detailed 24-h logs kept by the mothers (numbers of layers and arrangements of clothing and bedding as judged appropriate by mothers) when combined with the subsequent laboratory measurements of thermal resistance of the materials showed that the total thermal resistance of bedding plus clothing for the swaddled infants was significantly higher than for non-swaddled infants at 1 month and 3 months. This was reflected in the higher microenvironmental temperatures (inside the bedding but outside the clothing) of the swaddled versus non-swaddled infants. The core body temperature was also slightly but significantly higher in the swaddled group at 1 month, but not at 3 months of age. Despite these differences, however, the peripheral temperatures of the non-swaddled infants (a marker of peripheral vasoconstriction in response to cold stress) were not lower than those of the swaddled infants, at either age, suggesting that both groups of infants were in thermoneutral conditions. There were no episodes of increased peripheral temperature accompanied by rises in core temperature, which would have been suggestive of heat stress.

Room temperatures recorded in apartments in Ulaanbaatar were similar to those previously reported in the UK.^13^ ^15–17^ In a study in Bristol we recorded average indoor temperatures in infants’ bedrooms in winter of 18–19°C, and in the summer of 20–22°C,^13^ with less than 3°C difference between night-time and daytime temperatures, in summer and winter. Mongolian infants living in a Ger experienced greater room temperature variation in 1 day than UK infants experience over the course of a year with minimum temperatures sometimes as low as 0°C and maximum temperatures up to 25°C.

In this extreme environment, potential adverse consequences might have been anticipated, such as hypothermia at night or hyperthermia in the daytime. However, core body temperatures were remarkably similar in traditional Gers and apartments at both ages, whether the infants were swaddled or in sleeping-bags. On an individual level there were no recorded episodes of significant hypothermia or hyperthermia (core temperatures below 35°C or above 38°C, respectively).

A marked fall in core temperature during the night-time was observed for 3-month-old infants in sleeping-bags living in Gers. This fall in core temperature at this age is expected^16–20^ and was accompanied by a rise in peripheral temperature, showing that it was not an effect of cold stress (which leads to peripheral vasoconstriction and thus a fall in peripheral temperatures) but rather to the normal process previously reported in infants from around this age: that of active peripheral vasodilatation with increased heat loss leading to a fall in core temperature during the early part of the night-time hours, and with a reversal of this process during the latter part of the night.^15–22^ The indoor temperature fell, but the core temperature fall was independent of the minimum indoor temperature, which commonly occurred at the time core infant temperature was rising. Thus, the greater fall in the core temperature of infants in sleeping-bags reflects the development of a normal diurnal pattern of temperature changes with age. This diurnal pattern of temperature change, exactly as previously described in infants in studies in the UK,^15–21^ ^23^ commonly becoming clearly marked by around 3 months of age, was not seen in this study in infants who were swaddled.

A range of factors, including preterm birth, low birth weight, low Apgar scores and bottle-feeding have been reported to be associated with slightly delayed development of diurnal temperature patterns in infancy.^15^ ^22^ ^23^ None of these factors were present in our sample of infants and thus cannot explain the differences between the diurnal temperature patterns seen in the infants in the present study who were swaddled compared with those in sleeping-bags. Indeed the median birth weight of the infants in sleeping-bags was by chance 300 g less than for the swaddled infants, which might have been expected to contribute to later maturation of diurnal temperature pattern in these infants. We found no evidence of a relationship between birth weight and infant temperature in our data.

There were several strengths and weaknesses in our study. The unique set-up of this study enabled investigations into thermal conditions that are difficult to create in the lab or other natural settings. The RCT design of the main study enabled the comparison of similar populations only different in their habitual swaddling or sleeping-bag type of clothing in a powerful study design. The study randomisation process and stratified subsampling ensured that the two study groups (swaddled and sleeping-bags) were similar in terms of the type of dwelling, crowding and maternal parity and all other characteristics. Our findings are generalisable to infants born healthy in Ulaanbaatar as the mothers were representative of the greater population, and in fact of all such healthy-at-birth infants subject to similar environmental and clothing conditions since this cohort of Mongolian infants were not exposed to other unusual circumstances. However, the small numbers involved in this thermoregulatory study did not enable multivariate analysis investigating other influences on thermoregulation in this population.

We tried to ensure that all infants were wrapped in bedding and clothing of similar total thermal resistance (‘tog values’), but were not entirely successful as the choice of clothing was left ultimately to the parents. The clothing plus sleeping-bags used were designed to be of approximately equal thermal resistance to the commonly used swaddling materials; and the study provided an opportunity to evaluate this low-cost, safe, locally sourced alternative approach to infant insulation and care in this extreme environment. Qualitative data from the study parents indicated that parents of infants using the sleeping-bags tried to create the same level of warmth for their infants with non-swaddling clothes and sleeping-bags/blankets, but their judgements did not match the insulation values they thought swaddling would provide since tog values in swaddled infants were consistently higher (by around 4 togs). We have previously shown that parents are skilled at achieving appropriate levels of added bedding and clothing to avoid cold stress,^13^ and this study confirms this observation—even in the extreme climate of Mongolia.

Total thermal resistance during the night-time was consistently 2–5 togs higher than during the daytime at both ages. This is in general agreement with other studies that infants were kept warmer during the night.^13^ ^15^ ^16^ ^20^ Despite heavy insulation, swaddling offered no identifiable thermal advantage or disadvantage to the baby over the use of a sleeping-bag at night.

Additionally, traditional sleeping arrangements certainly contributed to higher thermal insulation at night. Ninety-eight percent of infants slept in bed with one or both parents, under the parents’ bedding as well as the sleeping-bags or swaddling. It is possible that habitually high levels of insulation, with consequently much higher environmental temperatures for these infants with a higher core temperature in the daytime, may contribute to a delay in the development of the normal overnight fall in core temperature. This phenomenon has not previously been described, but previous studies^13–16^ have used much lower levels of thermal insulation. The present study of infants sleeping in their normal swaddling environment compared with our intervention, identified conditions that were far more extreme than would have been ethical to impose upon infants in an intervention study—either in the home or the laboratory.

Although there was no evidence that individual infants suffered significant cold stress or heat stress the microenvironment around swaddled infants was consistently significantly warmer than for those in sleeping-bags.

In conclusion, this study is unique in investigating infants’ thermal balance during early infancy, in naturalistic settings and in a very cold indoor environment, where traditional practices are well represented and within a culture in which studies have not previously been conducted. Furthermore, the study addresses a very important issue (temperature maintenance) in the care of infants in a harsh climate, in Mongolia and in many low-income and middle-income countries where traditional swaddling remains a central infant care practice, commonly interacting with changing housing arrangements.

Overall, there is evidence that traditional Mongolian swaddling of healthy infants is not associated with identifiable thermal disadvantages or advantages at 1 month and 3 months of age. It seems however that, for swaddled infants in apartments, less wrapping may be needed.

The observed differences in the development of the nocturnal fall in core temperature between swaddled infants and those in sleeping-bags gives an interesting insight into developmental physiology. The significance of the wide normal variation in the development of this pattern is not known but might stimulate further research.

## Supplementary Material

Web supplement
