# Supplementary material for: Thermoregulatory effects of swaddling in Mongolia: a randomised controlled study
Source: Arch Dis Child. 2015 Oct 29;101(2):152–60. doi: 10.1136/archdischild-2014-307908 (PMC4752649; doi:10.1136/archdischild-2014-307908)

Appendix I. a) A one month old swaddled infant; b) A one month old baby in the cotton sleeping bag given to families. Note: Cots were very rarely used and only during day-time for placing the baby in a safe environment. At the time of the study in Mongolia, there were very few quality and affordable warm baby clothing options in shops. In order to facilitate families randomised in the non-swaddling group (intervention arm of the trial) to keep their babies warm in the extreme winter and to separate the effects of high thermal resistance of bedding from the effects of tight swaddling, we designed 2 infant sleeping bags which covered the baby from neck to feet, including the arms. We provided one each of cotton and sheepskin sleeping bags plus locally sewn cotton one-piece suits to the non-swaddling families (hereafter referred to as sleeping bag group) and some cotton sheets and commonly used blankets for the swaddling group. We designed the infant sleeping bags with the intention that, when used with 2-3 layers of cotton baby clothes they would be of approximately equal thermal resistance to swaddling materials commonly used. Families using the infant sleeping bags were advised to dress the baby in 2 or more layers of cotton onepiece suits, and then to use the cotton sleeping bag and additionally, the sheepskin sleeping bag if they felt this was necessary. c) A ger: An original home for nomadic herders, it is easy to dismantle and move, made from a wooden frame covered by several layers of wool felt more than 2cm thick and heated by a central stove with a chimney that is also used for cooking.

a)

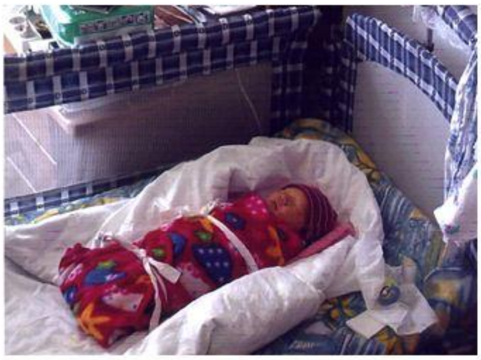

b)

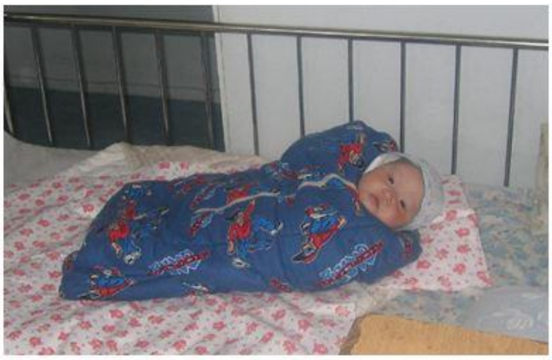

c)

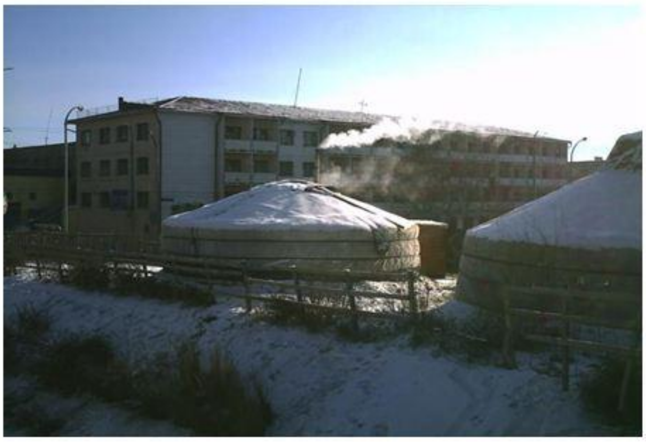

Appendix II. Median room temperatures at one and three months for infants living in apartments (red) and in gers (blue)

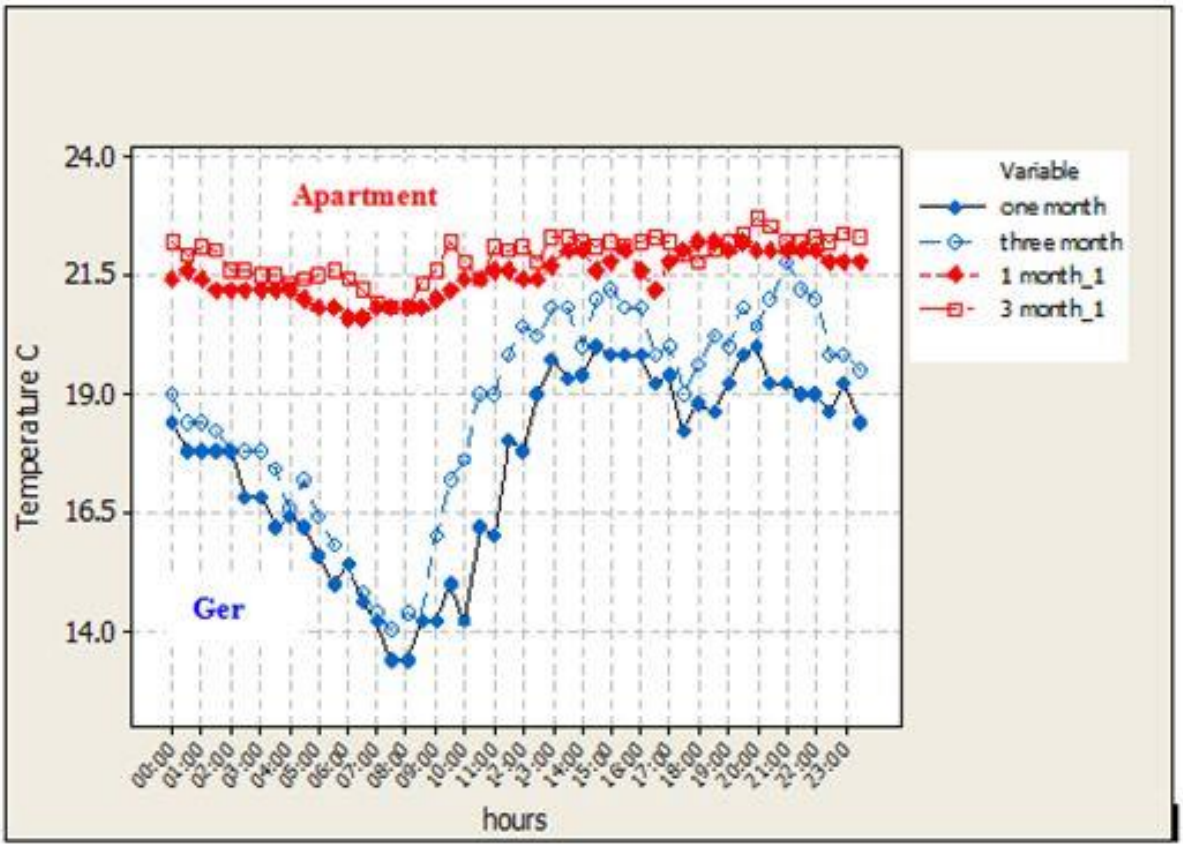

Appendix III. Total thermal resistance (tog values) of bedding and clothing for 1 month old swaddled and sleeping bag infants living in: a) gers; and b) apartments

a)

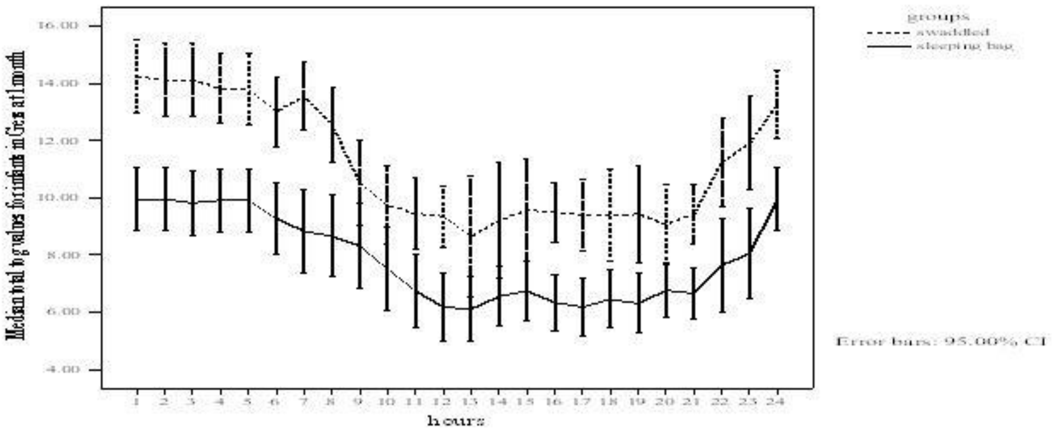

b)

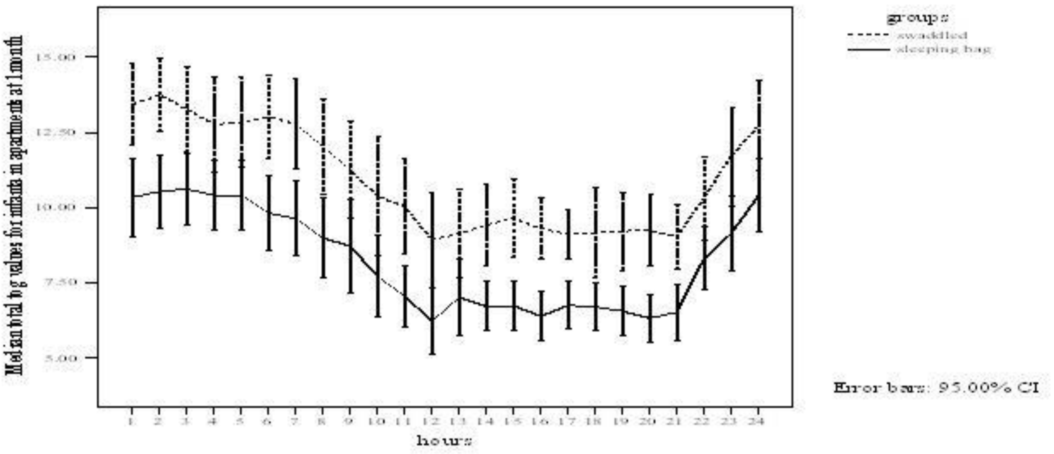

Supplement: Web supplement [file archdischild-2014-307908-s1.pdf]
